# Proof of mechanism investigation of Transcutaneous auricular vagus nerve stimulation through simultaneous measurement of autonomic functions: a randomized controlled trial protocol

**DOI:** 10.1186/s13030-024-00311-x

**Published:** 2024-06-18

**Authors:** Ruri Katsunuma, Tsunehiko Takamura, Mitsuhiko Yamada, Atsushi Sekiguchi

**Affiliations:** 1 Department of Neuropsychopharmacology, National Institute of Mental Health, National Center of Neurology and Psychiatry, Kodaira, Tokyo Japan; 2 School of Psychology, Faculty of Health and Behavioural Sciences, The University of Queensland, Brisbane, Australia; 3 Department of Behavioral Medicine, National Institute of Mental Health, National Center of Neurology and Psychiatry, Kodaira, Tokyo Japan; 4 Department of Pathophysiology, Faculty of Human Nutrition, Tokyo Kasei Gakuin University, Chiyoda-ku, Tokyo, Japan

**Keywords:** HRV, MRI, Neuromodulation, Parasympathetic, Sympathetic, Nucleus tractus solitarius

## Abstract

**Background:**

The autonomic nervous system plays a vital role in regulating physiological functions. Transcutaneous auricular vagus nerve stimulation (taVNS) is a method that provides insights into autonomic nerve modulation. This paper presents a research protocol investigating proof of mechanism for the impact of taVNS on autonomic functions and aims to both deepen theoretical understanding and pave the way for clinically relevant applications.

**Methods:**

This protocol employs a single-blind, randomized cross-over design involving 10 healthy male participants. Simultaneous assessment of both the afferent and efferent aspects of the vagus nerve will be performed by integrating physiological measures, magnetic resonance imaging, and a questionnaire survey. Electrocardiogram will be measured to assess changes in heart rate, as a primary outcome, and heart rate variability. Active taVNS and sham stimulation will be compared, which ensures precision and blinding. Electrical stimulation will be applied to the left *concha cymba* and the left *lobule* for the active and sham conditions, respectively. The specific parameters of taVNS involve a pulse width of 250 µs, a frequency of 25 Hz, and a current adjusted to the perception threshold (0.1 mA ≤ 5 mA), delivered in cycles of 32 s on and 28 s off.

**Conclusions:**

This research investigates proof of mechanism for taVNS to elucidate its modulatory effects on the central and peripheral components of the autonomic nervous system. Beyond theoretical insights, the findings will provide a foundation for designing targeted neuromodulation strategies, potentially benefiting diverse patient populations experiencing autonomic dysregulation. By elucidating the neural mechanisms, this study contributes to the evolution of personalized and effective clinical interventions in the field of neuromodulation.

**Trial Registration:**

JRCT, jRCTs032220332, Registered 13 September 2022; https://jrct.niph.go.jp/latest-detail/jRCTs032220332.

## Background

The autonomic nervous system (ANS) plays an indispensable role in various physiological functions, including cardiovascular activity, visceral perception, and emotional states [1]. Maintaining homeostasis relies on the delicate balance between the sympathetic and parasympathetic branches of the ANS [2]. Transcutaneous auricular vagus nerve stimulation (taVNS) has emerged as a cutting-edge tool to elucidate the mechanisms of autonomic regulation [3]. As a linchpin of the parasympathetic system, the vagus nerve’s extensive connections with the central nervous system positions it at the core of autonomic control [4]. Recent advancements underscore the potential of taVNS to modulate autonomic activity and influence functions such as heart rate and heart rate variability (HRV) [5, 6]. To gain a more comprehensive understanding, it is imperative to integrate these validated insights by examining indices of the afferent (e.g., brain magnetic resonance imaging [MRI]) and efferent (e.g., heart rate, pupil diameter) branches.

Offering a non-invasive approach with therapeutic potential, taVNS holds promise for addressing autonomic dysregulation [3]. The present study employs a comprehensive approach that integrates physiological, neural, and perceptual assessments to unravel the intricate influence of taVNS on autonomic functions, bridging the gap between theoretical understanding and clinical application. The study aims to investigate the proof of mechanism (POM) behind taVNS, focusing specifically on heart rate as a primary outcome and HRV and brain MRI as key indicators of vagal influence on the efferent and afferent tracts, respectively. Building on prior findings, this study investigates the immediate effects of taVNS intervention on autonomic functions through the use of a stimulus variable known for heart rate modulation.

This comprehensive assessment includes physiological measures, functional MRI (fMRI), and perceptual evaluations and provides a nuanced understanding of the impact of taVNS on autonomic pathways. fMRI is a reliable and complementary physiological assessment of afferent response, and by exploring changes in brain connectivity, it sheds light on the neural underpinnings of taVNS [7] by providing a reliable index to investigate the effects of taVNS modulation through the afferent branch. This holistic approach positions our study at the forefront of research and aims to establish a solid measurement of parasympathetic response though vagus nerve stimulation. Specifically, we seek to elucidate the association between the vagus nerve and autonomic functions, which has valuable implications for clinical applications and advancing our understanding of the neural mechanisms underlying autonomic regulation.

A previous study reported significant reductions in heart rate following taVNS stimulation in 15 healthy participants [5]. In another study, changes in HRV were observed in 13 healthy participants [6]. However, these changes were transient, lasting only 30–60 s, and thus lacked sustained effects. Notably, these studies used mixed-gender data, employed various stimulus variables, and were designed to assess the strength of effects on heart rate. Robust short-term decreases in heart rate and alterations in HRV immediately post-stimulation were consistently observed across all stimulus variables.

In the present study, we aim to enhance the specificity of our investigation by adopting a stimulus variable known to induce a more potent effect on heart rate reduction (25 Hz). Focusing on the short-term effects as the primary evaluation criterion, we anticipate detecting a reduction in heart rate in 10 cases by focusing on male subjects to exclude the effect of differences by sex. Because empirical evidence has demonstrated the impact of the menstrual cycle on the vagal nerve system [8, 9], this strategic approach of restricting the participants to men allows for a more nuanced understanding of the immediate impact of taVNS on heart rate dynamics by controlling the potentials that confounds such effects. Therefore, in this study, we will conduct a sham-controlled, single-blind randomized controlled trial targeting a healthy population, with the aim of pinpointing the POM for taVNS. The adoption of a single-blind study design ensures that participants remain unaware of whether they are receiving actual taVNS stimulation or a placebo intervention. This rigorous methodology was chosen to provide a thorough insight into the mechanisms underlying the effects of taVNS in a healthy population.

## Methods

### Participants

Ten healthy male participants will be recruited via internal advertisement at the institute where the research is to take place. The inclusion criteria include healthy males aged 20–39 years. Exclusion criteria are smokers, individuals with regular medication intake, a history of glaucoma or cerebrospinal fluid shunt surgery, and auricular hematoma (to facilitate the placement of taVNS electrodes). Participants will also be screened for MRI exclusion criteria, including nonremovable metal parts in/on the body, tattoos, known neurological diseases, and claustrophobia. Eligibility and exclusion criteria will be assessed through a physician interview. Participants deemed eligible have already been approached for written consent.

### Procedures

#### Experimental design

A single-blind randomized cross-over design will be used. Random assignment will be performed using a computer-generated randomization system by an institutional Academic Research Organization section to determine whether the participants would be assigned to the target or sham session first.

#### Baseline assessment (pre)

The participants will undergo baseline assessments before the stimulation phase: will include a self-report questionnaire, MRI (40 min), and measurement of physiological parameters: electrocardiogram (ECG); pupil diameter, 15 min; heartbeat perception task, 5 min.

#### Stimulation phase

Physiological parameters (ECG; pupil diameter. 15 min; heartbeat perception task. 5 min) will be measured under stimulation. MRI (rest, 15 min) will also be performed during stimulation.

#### Post-stimulation assessment

The assessment will continue without stimulation. Physiological parameters (ECG; pupil diameter, 15 min; and heartbeat perception task, 5 min) will be measured, MRI (rest, 15 min) will be performed, and a self-report questionnaire will be administered.

#### Cross-over evaluations

The same set of evaluations will be conducted at intervals of at least 1 week (details shown in the *Interval Considerations* section below). Stimulation methods will be crossed to account for any variations.

#### Adverse event evaluation

Participants will be instructed via email to contact the physician if they experience any adverse events during the experimental period (from the first test date to 1 week after the second test date).

#### Interval considerations

An interval of at least 1 week will be maintained to eliminate the potential carryover effect of taVNS (15-min stimulation). Previous studies employing crossover designs have utilized various stimulus intervals, including 50-min stimulation, 48-h interval [10]; 4-min stimulation, 1-week interval [11]; and 30-min stimulation, 1-week interval [12]. Based on the fact that prior studies set a maximum of 1 week to eliminate the carryover effect, we also set a 1-week interval in the present study, thereby aligning with previous practices.

### Heartbeat perception task

To examine the effects of the taVNS on changes in visceral perception, we will employ a heartbeat detection task. Participants will be instructed to independently count their heartbeats during specific intervals (25 s, 35 s, and 45 s) without relying on palpation, while their actual heart rate was concurrently monitored using a pulse meter (see [13] for details).

### Physiological measures

#### ECG

ECG will be measured using a two-lead ECG acquisition device (BrainAmp; Brain Products GmbH, Gilching, Germany) in order to assess changes in heart rate, as a primary outcome, and HRV. ECG data will be recorded using two disposable sticker-type electrodes, one on the left arm and one on the right arm, and another electrode on the right leg.

#### Pupil diameter

The variation in pupil diameter may reflect the activity of the nucleus tractus solitarius and the nucleus accumbens. Therefore, we will seek to evaluate the POM for the taVNS by analyzing the rate of change before and after stimulation. Pupil diameter was measured using a wearable eye-tracking device (Pupil Core; Pupil Labs, Berlin, Germany).

#### MRI acquisition

An MRI system equipped with a head coil (MAGNETOM Skyrafit 3T; Siemens Healthineers, Erlangen, Germany) will be used to acquire an anatomical volume image and fMRI images of each participant. To obtain a reference image for analysis, a structural image (T1-weighted magnetization-prepared rapid gradient-echo) will be captured with the following sequence parameters: repetition time (T_R_)/echo time (T_E_) = 2500/2.18 ms, voxel size = 0.8 × 0.8 × 0.8 mm, flip angle = 8°, and field of view (FOV) = 206 × 206 mm. To obtain resting-state fMRI images, parameters for single-shot echo-planar imaging will be set at T_R_/T_E_ = 800/34.4 ms, 60 axial slices with a thickness of 2.4 mm and a 0.6-mm interslice gap, flip angle = 52°, matrix size = 86 × 86, and FOV = 206 × 206 mm. Images will be angled along a line connecting the anterior and posterior commissures.

Our protocol aligns with the harmonized protocol for clinical MRI studies in the context of a national project, Brain/MINDS Beyond (https://brainminds-beyond.jp) to ensure standardized and high-quality neuroimaging. During resting-state fMRI, participants will be asked to keep their eyes open in order to ensure consistent data collection.

### Questionnaires

#### Multidimensional assessment of awareness of interoceptive awareness (MAIA)

The MAIA was designed to examine the interoceptive-perceptive characteristics of study participants. This self-report questionnaire consists of 32 items and assesses 8 dimensions of trait interoceptive sensitivity [13]. The current study will use the Japanese version of MAIA, which has been validated using exploratory factor analyses that resulted to reducing the questionnaire items from 32 to 25 and from 8 to 6 dimensions [14]. Despite these modifications, the items used in the current study will include the original 32-item version to ensure compatibility with data from previous research [15].

#### State-trait anxiety inventory (STAI)

The STAI was designed to examine mood changes in individuals undergoing taVNS. This self-report questionnaire consists of 40 items and measures state anxiety and trait anxiety. It uses a four-point Likert scale, with higher scores indicating greater anxiety [16]. The Japanese version of STAI will be used in the current study. Its reliability and validity have been confirmed [17].

#### Somatic symptom scale-8 (SSS-8)

The SSS-8 was designed to assess the physical symptoms of study participants. This self-report questionnaire consists of 8 items and assesses somatic symptom burden. It uses a five-point Likert scale, with higher scores indicating higher levels of somatic symptoms [18]. The current study will use the Japanese version of the SSS-8, which has been validated linguistically and psychologically and has been shown to have good internal consistency [19].

### Interventions

#### Active taVNS

Active taVNS will be performed using a tVNS®R stimulator (tVNS Technologies GmbH, Erlangen, Germany), an adaptation of the commercially available tVNS®L medical device in Europe tailored for clinical research with adjustable stimulation variables. tVNS®R is considered a medical device in Europe, which is pivotal in our study.

#### Stimulation protocols

##### Test Stimulation (taVNS stimulation)

Electrical stimulation will be applied to the left auriculovagal boat (an area innervated by the auricular vagus nerve).

##### Control stimulus (Sham Stimulation)

Electrical stimulation will be applied to the left *lobule*.

##### Stimulus variables

Pulse width: 250 µs, Frequency: 25 Hz, Current: perception threshold (0.1 mA ≤ 5 mA), 32 s on/28 s off.

Utilizing the stimulus variables of the tVNS®L medical device, a 15-min duration allows for a thorough examination of the effects of taVNS with minimal burden on the participant. The frequency of taVNS will be set to below the participants’ pain perception in both the target and sham stimulation trials.

##### Device application

An experimenter with knowledge of the allocation results will affix the electrodes to the participant’s auricular region just before the baseline examinations and the heartbeat perception task. Immediate covering of the participant ensures evaluator blinding, preventing other experimenters from observing the allocation results. This approach ensures precision in stimulation, control, and blinding, thereby maintaining the integrity of the study’s findings.

### Data analysis

#### Rate of change in heart rate

The rate of change in heart rate and pupil diameter before and after both the taVNS and sham stimulation will be calculated. A one-sample test will be performed for the difference between the two stimuli (during taVNS vs. during sham stimulation). The effects of stimulus order will also be evaluated by group.

#### HRV

As indices of HRV in the time domain, we will use the mean value of the R-R interval (i.e., the interval between R-wave peaks in the ECG), the standard deviation of the R-R interval, the root mean square of the difference between adjacent R-R intervals, and the number of heartbeats with an R-R interval of 50 ms or longer. The power spectral density of the R-R interval data will be analyzed, and the power in the low-frequency range (LF), power in the high-frequency range (HF), and the LF/HF ratio will be used as indices in the frequency domain. For HRV analysis, analysis of variance will be performed for two factors: (1) pre- and post-stimulus and (2) during taVNS stimulation.

#### Percentage change in pupil diameter

The rate of change in pupil diameter during taVNS stimulation and during sham stimulation will be calculated. A one-sample test will be performed to analyze the difference between the two stimuli (during taVNS stimulation vs. during sham stimulation). The effects of stimulus order will be evaluated by group.

#### Brain imaging data

A general linear model will be used to identify brain regions and circuits that show significant correlations with pre- and post-stimulus changes in each index during taVNS and sham stimulation. T1-weighted images will be used to identify the location of the nucleus tractus solitarius, locus coeruleus, anterior insular cortex, anterior cingulate cortex, and amygdala, serving as seeds to assess functional brain connectivity at rest. Resting-state functional brain connectivity will also be evaluated, using the anterior insular cortex as a seed. Statistical adjustments for age and other factors will be made when evaluating indices that differ between changes during taVNS stimulation and changes during sham stimulation. Additional analyses will be performed when deemed necessary or meaningful.

#### Behavioral and psychological measures

The relation between the rate of change in internal perception, physical symptoms, and psychiatric symptom scale as well as the rate of change during taVNS and sham stimulation will be evaluated using analysis of variance and statistical modeling.

To this end, the MAIA, STAI, and SSS-8 will be analyzed by performing a *t*-test for each total score and sub-score, by comparing taVNS and sham sessions. Additional analyses will also be performed when deemed necessary or significant.

## Discussion

This study aims to investigate a POM by simultaneously assessing both the afferent (fMRI) and efferent (HRV, pupil diameter) aspects of the vagus nerve, which will provide a distinctive advantage in comprehending the holistic effects of taVNS. Through the evaluation of the central projections via fMRI and autonomic responses via HRV and pupil diameter, our research provides a comprehensive view of ANS modulation [20–22]. This concurrent examination explores the dynamic interplay between the central and peripheral components, elucidating the intricacies of vagal stimulation on both the afferent and efferent pathways.

The simultaneous evaluation of vagal pathways provides a comprehensive understanding of the modulatory effects of taVNS on both the central and peripheral components of the ANS. Elucidating these effects of taVNS on the central and peripheral nervous systems, as demonstrated through concurrent evaluation of the vagal afferent and efferent pathways, will lay a foundation for designing targeted clinical trials. This will not only enable a more accurate evaluation of taVNS efficacy across patient cohorts but also ensure a standardized approach to assessing the utilization and effectiveness of taVNS stimuli in clinical studies. For instance, evidence obtained by simultaneous measurement of afferent and efferent response towards taVNS will enable us to further investigate the neural plastic effects of neuromodulation, establishing the POM as a cornerstone for practical applications and advancing personalized medicine.

Specifically, our findings will contribute to the growing body of evidence supporting the therapeutic potential of taVNS. Deepening our understanding of the neural mechanisms underlying taVNS will enable us to provide a robust assessment method for incorporating taVNS interventions across diverse patient populations, including those with epilepsy, tinnitus, depression, stroke, and Alzheimer’s disease [23]. Additionally, potential applications of taVNS in Parkinson’s disease and stress-related disorders may offer advantages through the enhancement of interoceptive functions [24, 25]. By clarifying the mechanisms of vagal stimulation responses, future clinical studies can leverage our methodology to provide tailored interventions for specific patient populations, enhancing the precision of other vagal stimulation interventions and exploring novel therapeutic applications.

In summary, the meticulous evaluation of the POM in the present study will form the foundation for mechanistic-based interventions, paving the way for the development of targeted neuromodulation strategies. The integration of our findings in clinical practice holds promise for improving patient outcomes across a wide range of medical conditions characterized by autonomic dysregulation. In navigating the evolving landscape of neuromodulation research, our study emphasizes the importance of a multifaceted approach in unraveling the complexities of the ANS, propelling us closer to personalized and effective clinical interventions.

## Data Availability

This manuscript has no associate data.

## Abbreviations

taVNS: Transcutaneous Auricular Vagus Nerve Stimulation
ANS: Autonomic Nervous System
HRV: Heart Rate Variability
MRI: Magnetic Resonance Imaging
POM: Proof Of Mechanism
fMRI: Functional Magnetic Resonance Imaging
ECG: Electrocardiogram
TR: Repetition Time
TE: Echo Time
FOV: Field Of View
MAIA: Multidimensional Assessment of Interoceptive Awareness
STAI: State-Trait Anxiety Inventory
SSS-8: Somatic Symptom Scale-8
LF: Low-Frequency Range
HF: High-Frequency Range
